# Verification of the Utility of Urinary L-FABP as a Predictor of Impaired Renal Function Based on Its Relationship with Changes in Renal Function

**DOI:** 10.3390/jcm15062243

**Published:** 2026-03-16

**Authors:** Yuichi Kato, Takeshi Sugaya

**Affiliations:** 1 National Hospital Organization Kumamoto Saishun Medical Center, 2659 Suya, Koshi 861-1196, Kumamoto, Japan; 2 Department of Nephrology and Hypertension, St. Marianna University School of Medicine, 2-16-1 Sugao, Miyamae Ward, Kawasaki 216-0015, Kanagawa, Japan; takeshi-sugaya@marianna-u.ac.jp

**Keywords:** urinary biomarkers, diabetic nephropathy, diabetic kidney disease, predictors of renal function decline, hypertension, tubulointerstitial disease, chronic hypoxia

## Abstract

**Background:** In patients with diabetes or hypertension, if appropriate intervention is not initiated early in the course of kidney disease, not only does the risk of progressing to end-stage renal failure increase, but mortality associated with vascular complications also rises as the disease progresses; therefore, there is an urgent need to develop urinary biomarkers that enable early diagnosis and prediction of disease progression. **Methods:** This two-year prospective observational study involved 185 outpatients. Patients were classified into two groups based on their baseline urinary L-FABP levels relative to the reference value of 8.4 μg/g·Cr at the start of the study. The rate of eGFR decline during the observation period was evaluated. **Results:** The results showed an interaction (synergistic effect) between urinary L-FABP and time in patients with diabetes or hypertension who had an eGFR of at least 60 mL/min/1.732 m^2^/kg/1.732 m^2^. Patients with high urinary L-FABP levels (>8.4 μg/g·Cr) exhibited a notably faster eGFR decline compared with those with low levels (≤8.4 μg/g·Cr). This finding suggests the potential of urinary L-FABP as a predictor of renal function decline; we evaluated this utility using the area under the ROC curve (AUC) and logistic regression analysis. The results indicate that urinary L-FABP holds potential as a predictor of renal function decline in diabetic or hypertensive patients with preserved eGFR. **Conclusions:** Among the analysis groups in which the validation was conducted, it was demonstrated that urinary L-FABP holds potential as a predictor of renal function decline in patients with diabetes or hypertension who have a maintained eGFR. Given that urinary L-FABP is thought to reflect tubulointerstitial damage associated with renal microcirculatory impairment, its future utility as a urinary biomarker for the early diagnosis and prognosis of chronic kidney disease (CKD) is anticipated.

## 1. Introduction

Among new dialysis patients in Japan, those with diabetic nephropathy (DN) have decreased in number in recent years [1]. However, a study of patients with type 2 diabetes found that more than 70% had atypical nephropathy, which is characterised by an eGFR below 60 mL/min/1.732 m^2^ without overt albuminuria [2]. In patients with atypical nephropathy, albuminuria is not a useful early diagnostic marker, which makes diagnosis difficult until the GFR actually declines. There is a need for urinary biomarkers that can enable diagnosis at an early stage when the GFR is still normal.

Liver-type fatty acid-binding protein (L-FABP) is expressed in the cytoplasm of human renal proximal tubule cells. It is believed to have a renoprotective function by transporting reabsorbed fatty acids and albumin into the nucleus. This process promotes β-oxidation in mitochondria and peroxisomes, thereby mitigating the cytotoxicity caused by lipid peroxidation [3,4,5]. Furthermore, the upstream transcriptional regulatory region of the L-FABP gene contains transcription factor binding sites associated with ischemia and lipid metabolism. Gene expression is induced in response to various ischemic and oxidative stress conditions [5,6]. In other words, urinary L-FABP reflects tubulointerstitial injury caused by a decreased oxygen concentration associated with impaired renal microcirculation in kidney disease in real time [6,7,8]. Its characteristics are distinct from those of existing urinary biomarkers, such as urinary protein, urinary albumin, NAG, and β2-MG, which reflect renal dysfunction outcomes.

Furthermore, reports indicate that tubulointerstitial lesions are more favourable prognostic markers for renal disease than glomerular lesions [9,10,11]. Previous studies suggest that urinary L-FABP may be a useful biomarker for predicting the onset, severity, and progression of nephropathy [12,13], highlighting the importance of urinary biomarkers reflecting tubulointerstitial damage.

Meanwhile, Li Zhang et al. conducted a meta-analysis on the performance of urinary L-FABP in nephropathy. They proposed that urinary L-FABP could serve as a highly sensitive marker for the early diagnosis of nephropathy and for estimating its progression and severity. However, they noted that most of the included studies were small-scale, cross-sectional studies, and that larger, prospective, longitudinal studies with longer follow-up periods are needed to verify the utility of urinary L-FABP in diabetic patients [14].

In this study, we evaluated the utility of urinary L-FABP as a predictor of renal function decline by classifying 185 patients into two groups based on their urinary L-FABP levels at baseline and assessing the relationship between urinary L-FABP and eGFR changes through a 2-year prospective observational follow-up study.

## 2. Materials and Methods

This two-year prospective observational follow-up study was conducted from October 2015 to September 2017. It examined outpatients who were not receiving renal replacement therapy at the National Hospital Organization Kyushu Medical Centre. The study was approved by the Ethics Committee of the National Hospital Organization Kyushu Medical Centre.

### 2.1. Study Population Size

The study population comprised 185 outpatients (all cases) who visited the Kyushu Medical Centre, underwent blood and urine tests, and had estimated glomerular filtration rate (eGFR) values that could be extracted from their electronic medical records.

The analysis groups validated in this study were derived by extracting all cases (*n* = 185) and cases with a baseline eGFR ≥ 60 mL/min/1.73 m^2^ (normal eGFR cases, *n* = 143) and then classifying them into two groups. Furthermore, patients who visited the diabetes or hypertension department were extracted from all cases and normal eGFR cases and classified into two etiological groups as the high-risk group. The etiology groups were composed as follows: In the overall high-risk group (*n* = 79), there were 39 Diabetes Internal Medicine (T2DM) cases and 40 Hypertension Internal Medicine (HTN) cases. In the normal eGFR high-risk group (*n* = 55), there were 29 Diabetes Internal Medicine (T2DM) and 26 Hypertension Internal Medicine (HTN) cases. Validation was performed across all four of these analysis groups.

### 2.2. Definitions

In order to examine the relationship between urinary L-FABP levels and eGFR progression, the subjects were categorised into two groups according to their baseline urinary L-FABP levels in relation to the reference value (8.4 μg/g·Cr), low (low urinary L-FABP group: ≤8.4 μg/g·Cr) and high (high urinary L-FABP group: >8.4 μg/g·Cr), which was defined as the L-FABP category.

Furthermore, to assess the rate of eGFR decline, a regression line was created using linear regression based on eGFR values at one and two years after baseline. The slope of this line was calculated as the annual change in eGFR using the SLOPE function and is termed the ‘eGFR slope’. To evaluate the utility of urinary L-FABP as a predictor of renal function decline, we defined ‘rapid decliner’ as a case where the eGFR slope exceeded a decline of 5 mL/min/1.73 m^2^/year, in accordance with the ‘(2012) KDIGO CKD Clinical Practice Guidelines’ [15]. The primary endpoint of renal function decline was then defined as the occurrence of rapid decliner status, and its relationship with urinary L-FABP was examined.

### 2.3. Urine Collection and Analysis

Ten millilitres of urine was collected from the subjects as part of their routine clinical care. The samples were then centrifuged at 2000 rpm for 10 min at room temperature. Urinalysis, urinary L-FABP, urinary albumin, and urinary creatinine were measured in the Biochemistry and General Testing Laboratory at Kyushu Medical Centre. Urinary L-FABP levels were quantified using Nordia^®^ L-FABP (Sekisui Medical, Tokyo, Japan), following the manufacturer’s instructions. The eGFR used for the analysis was calculated based on Japanese Society of Nephrology guidelines: 194 × serum creatinine (mg/dL) − 1.094 × age (years) − 0.287 for men and 0.739 × the male formula for women.

### 2.4. Statistical Analysis

Continuous variables that followed a normal distribution were expressed as the mean ± standard deviation (SD). Those not following a normal distribution were expressed as the median (interquartile range). Nominal variables were expressed as a percentage. The normality of the distribution was assessed using the Shapiro–Wilk test. Urine L-FABP and urine albumin were adjusted for urine creatinine. Non-normally distributed variables were log-transformed prior to statistical analysis.

Statistical analysis was performed using EZR (Easy R:64-bit Jichi Medical University Tochigi Prefecture). For comparisons between two groups, a Student’s *t*-test was used for normally distributed continuous variables, and a Mann–Whitney U test was used for non-normally distributed continuous variables. Fisher’s exact test was used for nominal variables. The effect of urinary L-FABP on longitudinal changes in eGFR during the observation period was analysed using repeated-measures ANOVA with the L-FABP category. Next, the diagnostic accuracy of urinary L-FABP in identifying rapid decliners was evaluated using the area under the receiver operating characteristic curve (ROC curve area: AUC). Furthermore, the utility of urinary L-FABP as a predictor of rapid decliner status was evaluated using univariate and multivariate (logistic regression) analyses adjusted for clinical parameters (urinary L-FABP, urinary albumin, eGFR2015, gender, and age). In all analyses, *p*-values < 0.05 were considered statistically significant.

## 3. Results

### 3.1. Patient Characteristics and Annual Change in eGFR

Table 1 shows the baseline characteristics of the patients at the start of the observation period, as well as the change in eGFR over two years (eGFR slope), for the analysis group.

Comparisons between all cases and normal eGFR cases with their respective high-risk groups showed significant differences in eGFR at baseline, 1 year, and 2 years. Furthermore, a significant difference in gender was observed when comparing all cases with the high-risk group. Although no clear significant difference in eGFR slope was observed between the analysed groups, the normal eGFR cases (high-risk group) had the largest slope (−4.2 mL/min/1.73 m^2^).

### 3.2. Comparison of eGFR Across L-FABP Categories

Table 2 shows the results of the comparison of eGFR across L-FABP categories at one and two years after the start of the observation period for the analysis group.

No significant difference was observed between L-FABP categories at baseline in all cases and in normal eGFR cases. However, after 2 years, a significant difference in eGFR was observed between L-FABP categories, with significantly lower eGFR values in the L.category-High group compared to the L.category-Low group. Furthermore, in all cases (high-risk group) and normal eGFR cases (high-risk group), no significant differences in eGFR were observed between L-FABP categories at any time point over the 2-year period. However, in both analysis groups, baseline eGFR was higher in the L.category-High group than in the L.category-Low group. However, after 2 years, this pattern reversed, with eGFR being higher in L.category-Low than in L.category-High.

Plotting the eGFR trends for both analysis groups revealed that the L.category-High group showed a steeper negative slope on the graph, indicating the rate of eGFR decline compared with the L.category-Low group.

#### 3.2.1. Comparison of eGFR Slope Across L-FABP Categories

Table 3a presents the results of a statistical analysis comparing eGFR slope over a two-year period across L-FABP categories within the analysis group.

In all analysis groups, the L.category-High group showed a greater negative eGFR slope and faster rate of eGFR decline compared to the L.category-Low group. Statistical comparisons of eGFR slope between L-FABP categories revealed significant differences in eGFR slope between L-FABP categories in all cases (high-risk group) and in normal eGFR cases (high-risk group) (*p* < 0.05) between L-FABP categories. In both groups, L.category-High showed a significantly steeper negative slope and a significantly faster rate of eGFR decline compared to L.category-Low. Furthermore, the difference in the rate of eGFR decline was more pronounced in the normal eGFR cases (high-risk group) than in all cases (high-risk group).

#### 3.2.2. Effect of L-FABP Category on eGFR Changes over Time

Table 3b presents the findings of the repeated-measures ANOVA analysis investigating the impact of L-FABP category on eGFR changes over time.

A significant decline in eGFR was observed over time across all analysis groups (*p* < 0.001 for time). An interaction effect (a synergistic effect arising from the mutual influence of two factors) was observed between the L-FABP category and time in the total cases (high-risk group), normal eGFR cases (high-risk group), and normal eGFR cases (factor 1.L.category: time *p* < 0.05). In other words, within these analytical groups, changes in eGFR over time differed between L-FABP categories due to the synergistic effect of urinary L-FABP and time. This revealed that the rate of eGFR decline was significantly faster in the L.category-High group compared to the L.category-Low group.

### 3.3. Diagnostic Accuracy of Rapid Decliners Based on Urinary L-FABP

Table 4 shows the diagnostic accuracy for rapid decliners based on the area under the ROC curve (AUC) of the following clinical parameters: urinary L-FABP, urinary albumin, eGFR2015, and age. These parameters were analysed in the analysis group.

Urinary L-FABP demonstrated moderate diagnostic accuracy for identifying rapid decliners in all cases (high-risk group) and in normal eGFR cases (high-risk group). The primary area under the curve parameter for all cases (high-risk group) was eGFR2015 (AUC: 0.697), while the secondary major parameter was urinary L-FABP (AUC: 0.639). In normal eGFR cases (high-risk group), the primary large-area parameter was urinary L-FABP (AUC: 0.695), and the secondary large-area parameter was urinary albumin (AUC: 0.649). Urinary L-FABP demonstrated superior diagnostic accuracy for rapid decliners within the normal eGFR cases (high-risk group) subgroup.

### 3.4. Evaluation of the Utility of Urinary L-FABP as a Predictor for Rapid Decliners

Table 5 shows the results of the evaluation of predictors for rapid decliners in the analysis group using both univariate and multivariate analyses. The dependent variable was the presence or absence of rapid decliners, and the independent variables were the clinical parameters.

In all cases and in the normal eGFR group, urinary albumin and eGFR2015 were predictive factors for rapid decliners in the univariate analysis and multivariate analysis adjusted for clinical parameters. In all cases (high-risk group), eGFR2015 was a predictive factor for rapid decliners in the univariate analysis. However, no association was found between urinary L-FABP and rapid decliners. In contrast, in the normal eGFR cases (high-risk group), urinary L-FABP was shown to be a significant predictor of rapid decliners in the univariate analysis (Odds Ratio, 1.15; 95% CI, 1.030–1.280: *p* = 0.014). However, no association with rapid decliners was observed in the multivariate analysis after adjusting for clinical parameters. However, in a multivariate analysis excluding urinary albumin from the clinical parameters, urinary L-FABP was shown to be a predictor of rapid decliners (odds ratio, 1.15; 95% CI, 1.030–1.290; *p* = 0.017).

## 4. Discussion

This study examined the potential of urinary L-FABP as a predictor of renal function decline by investigating its relationship with urinary L-FABP levels and estimated glomerular filtration rate (eGFR) changes through cross-sectional and longitudinal research. The results revealed that in patients with diabetes or hypertension and an eGFR of at least 60 mL/min/1.732 m^2^, the impact of urinary L-FABP on eGFR change differed based on baseline urinary L-FABP levels. A significantly faster eGFR decline was observed in patients with high urinary L-FABP levels. Furthermore, in the same patient, urinary L-FABP demonstrated superior diagnostic accuracy for renal impairment compared to other clinical parameters, indicating its potential as a predictive factor for renal dysfunction.

A clinical trial retrospectively divided non-diabetic chronic kidney disease (CKD) patients into progressive and non-progressive groups after one year of observation, evaluating clinical markers. The study found that urinary L-FABP levels were significantly higher in the progressive group than in the non-progressive group [16]. A five-year prospective observational study of stable non-diabetic hypertensive patients found that those with urinary L-FABP levels above the upper limit (8.4 μg/g·Cr) were more likely to experience a decline in renal function than those within the normal range [17]. Furthermore, prospective observational studies in patients with type 1 and type 2 diabetes have reported that elevated urinary L-FABP levels are associated with an increased incidence of renal outcomes, such as increased urinary albumin excretion and a decreased eGFR. This demonstrates the excellent utility of L-FABP as an independent predictor of nephropathy progression [12,13].

In this study, urinary L-FABP demonstrated significantly accelerated eGFR decline in patients with diabetes or hypertension who maintained a stable eGFR, particularly in the high urinary L-FABP group. Compared to other clinical parameters, it showed superior diagnostic accuracy for renal function decline and holds potential as a predictive factor for renal deterioration. These findings are consistent with previous studies evaluating the clinical utility of urinary L-FABP. We consider urinary L-FABP to be a useful clinical biomarker for suppressing the progression of renal failure by enabling the identification of patients at high risk for kidney disease progression.

Previous studies have reported a negative correlation between urinary L-FABP and eGFR [18,19]. However, this study found that patients with diabetes or hypertension had a higher baseline eGFR in the group with high urinary L-FABP levels than in the group with low urinary L-FABP levels. This seemingly contradictory phenomenon is presumed to be due to an increased albumin load on the proximal tubules caused by hyperfiltration in the high urinary L-FABP group at baseline, which leads to elevated urinary L-FABP. After two years, however, this trend reversed, with the low urinary L-FABP group exhibiting a higher eGFR than the high group. Analysis revealed that the rate of eGFR decline was significantly faster in the high urinary L-FABP group than in the low group, suggesting that factors accelerating eGFR decline exist in the high urinary L-FABP group during the hyperfiltration state. The renin–angiotensin system (RAS) has been associated with the development of hyperfiltration [20]. It has been reported that RAS activation plays a central role in the progression of kidney disease by inducing proteinuria, inflammation, the formation of intracellular reactive oxygen species, and hypoxia (ischemia) [21,22]. In other words, it is thought that these factors accelerating the rate of eGFR decline exist as multiple factors in the high-urinary-L-FABP group. By reflecting the influence of these factors accelerating kidney damage, urinary L-FABP is expected to serve as a clinical biomarker enabling the early diagnosis and treatment of kidney disease, as well as monitoring kidney disease and narrowing down the risk of progression. However, there is still insufficient research to verify the association between urinary L-FABP and the factors that accelerate renal impairment. We believe that a greater accumulation of clinical studies involving a larger number of cases over a longer period is necessary.

In recent years, there has been an increase in atypical diabetic kidney disease, leading to the proposal of DKD (diabetic kidney disease) as a comprehensive disease concept that encompasses both typical and atypical forms of nephropathy [23]. In atypical diabetic kidney disease, albuminuria is not a useful early diagnostic marker, which makes diagnosis difficult until GFR actually declines. Therefore, diagnostic methods for the early stage when the GFR is still normal are required.

Furthermore, studies analysing kidney tissue samples from patients with diabetes and hypertension report that, even in the early stages when eGFR is normal, histological interstitial and vascular lesions are often present. This suggests that eGFR underestimates the extent of kidney damage in the early stages of diabetes and hypertension [24,25].

This may also explain why eGFR at baseline did not demonstrate predictive utility for renal decline in patients with diabetes or hypertension whose eGFR remained normal throughout the study. These findings highlight the urgent need for additional biomarkers to complement urinary albumin and eGFR in identifying early renal impairment in patients with diabetes or hypertension.

This study demonstrated that urinary L-FABP holds potential as a predictor of renal function decline in patients with diabetes or hypertension who maintained a stable eGFR. This finding confirms the utility of incorporating urinary L-FABP into routine clinical practice alongside urinary albumin and eGFR for suppressing the progression of kidney damage at an early stage in patients with diabetes or hypertension. Furthermore, studies indicating that tubulointerstitial lesions are a more significant predictor of renal prognosis than glomerular lesions provide further evidence to support the effectiveness of using urinary L-FABP to suppress the progression of kidney disease [9,10,11].

Chronic hypoxia has been identified as a factor in the onset and progression of tubulointerstitial injury, which plays a key role in the progression of kidney disease [26,27,28]. Atsuko Kamijo-Ikemori et al. proposed that chronic hypoxia plays a dominant pathogenic role in triggering and accelerating the progression of early nephropathy. They hypothesised that urinary excretion of L-FABP might increase due to tubulointerstitial damage caused by chronic hypoxia, even in early nephropathy without albuminuria derived from glomerular damage [29]. Furthermore, studies evaluating the clinical significance of urinary L-FABP report that it serves as a real-time indicator of tubulointerstitial injury that significantly reflects the degree of damage and can be considered as a marker for the early diagnosis of kidney disease or to indicate renal prognosis [30,31,32,33].

In a longitudinal study using BOLD (blood oxygen level-dependent) MRI, Sugiyama et al. reported that hypoxic conditions were observed in the renal parenchyma even in stages of atypical diabetic kidney disease with a normal eGFR. They found that this hypoxia was involved in inducing and exacerbating tubulointerstitial injury and that it served as an independent prognostic factor for DKD, suggesting the potential of functional MRI as an early diagnostic method for DKD [34]. In other words, urinary L-FABP is considered to reflect tubulointerstitial injury associated with hypoxia due to impaired renal microcirculation. Therefore, urinary L-FABP, similar to BOLD MRI measurement of renal tissue oxygenation, is thought to hold excellent potential as an early diagnostic method for DKD [35].

This study has several limitations, including its single-centre nature, small sample size, and potential for subject bias. Additionally, this was a short-term observational follow-up study with a two-year observation period. While the eGFR slope was used as an indicator of eGFR decline rate, it should be noted that, although a linear eGFR slope decline has been reported in patients with type 1 diabetes, there is no established evidence that the eGFR slope declines in the same linear manner in patients with type 2 diabetes or other diseases.

Furthermore, in the normal eGFR group (high-risk group), the multivariate analysis adjusted for clinical parameters did not reveal an association between urinary L-FABP and rapid decliners. However, multivariate analysis excluding urinary albumin from the clinical parameters indicated that urinary L-FABP was a predictor of rapid decliners. This is likely due to the influence of multicollinearity, as a correlation was observed between urinary L-FABP and urinary albumin (correlation coefficient: 0.56, *p*-value: <0.001). Further discussion is warranted regarding the loss of statistical significance for urinary L-FABP after adjusting for urinary albumin.

This study demonstrated the utility of urinary L-FABP as a predictor of renal function decline in patients with diabetes or hypertension who maintain preserved eGFR. We believe that stratifying the risk of kidney disease progression using urinary L-FABP in these patients at an early stage when eGFR is preserved holds promise for suppressing the progression to renal failure. Furthermore, as diabetic patients age, treatment periods are prolonged, and multidisciplinary treatment is increasingly adopted, while the number of patients with atypical diabetic nephropathy is expected to rise. We anticipate that evaluations of urinary L-FABP’s potential as an early diagnostic method for this condition will continue.

## 5. Conclusions

In patients with diabetes or hypertension who maintained a stable eGFR, those with urinary L-FABP exceeding the reference value (8.4 μg/g·Cr) exhibited a faster rate of eGFR decline. Urinary L-FABP demonstrated potential as a predictor of renal function decline in these patients.

Moving forward, clinical research is needed to accumulate evidence for urinary L-FABP as a biomarker of early renal impairment, which would complement urinary albumin and the eGFR.

## Figures and Tables

**Table 1:** Patient background and change in eGFR over observation period (eGFR slope) in the analysis group.

		All Cases (*n* = 185)	All Cases (High-Risk Group: *n* = 79)	Comparison Between Etiological Groups: *p*-Value
Urinary L-FABP (μg/g·Cr)		3.5 (2.2~6.3)	4.0 (2.4~9.3)	NS
Urinary Albumin (mg/g·Cr)		16.3 (9.3~33.6)	16.6 (9.3~30.6)	NS
eGFR in 2015 (at baseline)		77.0 ± 23.1	69.3 ± 20.8	0.01
eGFR in 2016 (one year later)		73.8 ± 23.3	64.9 ± 19.4	0.003
eGFR in 2017 (two years later)		67.6 ± 21.4	59.5 ± 18.6	0.004
Gender	Male	56 (30.3%)	35 (44.3%)	0.034
	Female	129 (69.7%)	44 (55.7%)	
Age		67 (58~74)	68 (58.5~74.5)	NS
eGFR slope (mL/min/1.73 m^2^/year)		−3.4 (−6.4~−1.6)	−3.9 (−5.8~−1.9)	NS
		**Normal eGFR Cases (*n* = 143)**	**Normal eGFR Cases (High-Risk Group:** ***n* = 55)**	**Comparison Between Etiological Groups** **: ** ***p*** **-Value**
Urinary L-FABP (μg/g·Cr)		3.5 (2.1~6.3)	4.2 (2.5~9.6)	NS
Urinary Albumin (mg/g·Cr)		14.5 (9.2~32.9)	16.6 (9.7~33.2)	NS
eGFR in 2015 (at baseline)		80.9 (71.7~93.7)	75.5 (65.7~89.0)	0.022
eGFR in 2016 (one year later)		79.3 (68.5~93.3)	71.7 (63.3~81.1)	0.005
eGFR in 2017 (two years later)		72.4 (62.5~83.9)	65.2 (57.1~75.9)	0.006
Gender	Male	40 (27.97%)	23 (41.82%)	NS
	Female	103 (72.03%)	32 (56.18%)	
Age		64 (55.5~72.5)	67 (56~74)	NS
eGFR slope (mL/min/1.73 m^2^/year)		−3.9 (−7.3~−1.6)	−4.2 (−7.1~−2.0)	NS

**Table 2:** Comparison of eGFR among L-FABP categories at each time point during the observation period.

	eGFR in 2015 (at Baseline)		eGFR in 2016 (One Year Later)		eGFR in 2017 (Two Years Later)	
	Mean ± Standard Deviation	*p*-Value	Mean ± Standard Deviation	*p*-Value	Mean ± Standard Deviation	*p*-Value
All Cases						
L.category-Low (*n* = 149)	78.28 ± 22.03	NS	75.52 ± 22.88	0.043	69.46 ± 20.38	0.013
L.category-High (*n* = 36)	71.81 ± 26.63		66.80 ± 24.03		59.70 ± 23.70	
All cases (High-Risk Group)						
L.category-Low (*n* = 56)	67.6 ± 17.2	NS	64.4 ± 18.3	NS	59.7 ± 17.2	NS
L.category-High (*n* = 23)	73.2 ± 27.7		66.1 ± 22.2		58.9 ± 22.0	
	**eGFR in 2015 (at Baseline)**		**eGFR in 2016 (One Year Later)**		**eGFR in 2017 (Two Years Later)**	
	**Median (Quartile)**	***p*** **-Value**	**Median (Quartile)**	***p*** **-Value**	**Median (Quartile)**	***p*** **-Value**
Normal eGFR Cases						
L.category-Low (*n* = 116)	81.0 (60.2~136.5)	NS	81.1 (47.8~148.7)	NS	74.9 (36.8~125.8)	0.022
L.category-High (*n* = 27)	78.9 (60.2~129.5)		76.1 (52.6~112.5)		67.3 (47.7~121.2)	
Normal eGFR Cases (High-Risk Group)						
L.category-Low (*n* = 37)	74.7 (64.9~81.6)	NS	71.6 (62.6~81.1)	NS	68.8 (57.9~78.7)	NS
L.category-High (*n* = 18)	80.3 (68.6~94.1)		74.7 (67.8~78.3)		63.2 (56.4~71.6)	

**Table 3:** (**a**) Comparison of eGFR slope between urinary L-FABP categories in the analysis group. (**b**) Effect of urinary L-FABP category on changes in eGFR over time.

(**a**)		
**Analysis Group**	**eGFR Slope (Interquartile Range): mL/min/1.73 m^2^/Year**	**eGFR Slope Comparison (*p*-Value)**
All Cases (*n* = 185)	L.category-Low: −3.2 (−6.0~−1.6)	NS
	L.category-High: −4.7 (−7.4~−1.6)	
All Cases (High-Risk Group: *n* = 79)	L.category-Low: −3.2 (−5.0~−1.7)	0.026
	L.category-High: −5.0 (−8.8~−3.5)	
Normal eGFR Cases (*n* = 143)	L.category-Low: −3.2 (−7.0~−1.6)	NS
	L.category-High: −5.0 (−8.7~−2.3)	
Normal eGFR Cases (High-Risk Group: *n* = 55)	L.category-Low: −3.2 (−5.9~−1.6)	0.005
	L.category-High: −6.4 (−9.9~−4.3)	
(**b**)		
**Analysis Group**	**Repeated-Measures ANOVA Analysis: *p*-Value**	
All Cases (*n* = 185)	Factor1.L.category	NS
	Time	<0.001
	Factor1.L.category; Time	NS
All Cases (High-Risk Group: *n* = 79)	Factor1.L.category	NS
	Time	<0.001
	Factor1.L.category; Time	0.01
Normal eGFR Cases (*n* = 143)	Factor1.L.category	NS
	Time	<0.001
	Factor1.L.category; Time	0.025
Normal eGFR Cases (High-Risk Group: *n* = 55)	Factor1.L.category	NS
	Time	<0.001
	Factor1.L.category; Time	0.008

**Table 4:** Diagnostic accuracy of rapid decliners based on the area under the curve (AUC) of clinical parameters.

Analysis Group		AUC	Threshold	Sensitivity	Specificity
All Cases (*n* = 185)	LFABP	0.545	2.9	0.719	0.43
	ALB	0.645	38.6	0.412	0.868
	2015eGFR	0.682	88.9	0.469	0.818
	Age	0.564	50	0.234	0.909
All Cases (High-Risk Group: *n* = 79)	LFABP	0.639	3.1	0.875	0.4
	ALB	0.41	11.2	0.429	0.647
	2015eGFR	0.697	66	0.75	0.582
	Age	0.463	74	0.417	0.727
Normal eGFR Case (*n* = 143)	LFABP	0.548	2.9	0.707	0.447
	ALB	0.678	38.6	0.426	0.87
	2015eGFR	0.625	95.4	0.379	0.882
	Age	0.509	50	0.241	0.882
Normal eGFR Cases (High-Risk Group: *n* = 55)	LFABP	0.695	12	0.429	0.941
	ALB	0.649	40.4	0.5	0.917
	2015eGFR	0.607	89	0.476	0.853
	Age	0.502	77	0.429	0.824

Rapid decliner: Drop in eGFR above 5 mL/min/1.72 m^2^/year ⇒ event occurrence.

**Table 5:** Evaluation of predictors for rapid decliners using univariate and multivariate analyses.

	All Cases		All Cases (High-Risk Group)	
	*p*-Value	Odds Ratio (95%CI)	*p*-Value	Odds Ratio (95% CI)
Unadjusted				
Urinary Albumin	0.006	1.01 (1.000~1.03)	NS	1.02 (0.995~1.04)
eGFR.2015	<0.001	1.03 (1.020~1.050)	0.008	1.04 (1.010~1.070)
Urinary L-FABP	NS	0.686 (0.325~1.45)	NS	1.01 (0.988~1.040)
Sex [T.1]	0.034	2.16 (1.060~4.420)	NS	1.49 (0.560~3.990)
Age	NS	0.979 (0.956~1.00)	NS	0.977 (0.939~1.02)
Adjusted				
Urinary Albumin	0.011	1.02 (1.000~1.030)	NS	1.01 (0.992~1.03)
eGFR.2015	<0.001	1.04 (1.020~1.060)	NS	1.02 (0.978~1.06)
Gender [T.1]	NS	1.54 (0.595~3.980)	NS	0.844 (0.197~3.63)
	**Normal eGFR Cases**		**Normal eGFR Cases (High-Risk Group)**	
	***p*** **-Value**	**Odds Ratio (95% CI)**	***p*** **-Value**	**Odds Ratio (95% CI)**
Unadjusted				
Urinary Albumin	0.012	1.02 (1.000~1.030)	NS	1.02 (0.994~1.040)
eGFR.2015	0.004	1.03 (1.010~1.050)	NS	1.02 (0.989~1.06)
Urinary L-FABP	NS	1.47 (0.631~ 3.400)	0.014	1.15 (1.030~1.280)
Gender [T.1]	NS	1.88 (0.863~4.110)	NS	1.28 (0.422~3.90)
Age	NS	0.993 (0.967~1.02)	NS	0.99 (0.945~1.04)
Adjusted				
Urinary Albumin	0.036	1.01 (1.000~1.030)	NS	1.01 (0.994~1.03)
eGFR.2015	0.002	1.05 (1.020~1.080)	NS	1.02 (0.965~1.07)
Urinary L-FABP	NS	0.953 (0.839~1.080)	NS	1.1 (0.882~1.36)

## Data Availability

The original contributions presented in this study are included in the article. Further inquiries can be directed to the corresponding author.
